# Photochemical Haze Formation on Titan and Uranus: A Comparative Review

**DOI:** 10.3390/ijms26157531

**Published:** 2025-08-04

**Authors:** David Dubois

**Affiliations:** 1 NASA Ames Research Center, MS 245-6, Moffett Field, CA 94035, USA; david.f.dubois@nasa.gov; 2 Bay Area Environmental Research Institute, Moffett Field, CA 94035, USA

**Keywords:** solar system, Titan, Uranus, giant planets, atmospheres, ion-neutral reactions, haze formation, astrochemistry

## Abstract

The formation and evolution of haze layers in planetary atmospheres play a critical role in shaping their chemical composition, radiative balance, and optical properties. In the outer solar system, the atmospheres of Titan and the giant planets exhibit a wide range of compositional and seasonal variability, creating environments favorable for the production of complex organic molecules under low-temperature conditions. Among them, Uranus—the smallest of the ice giants—has, since Voyager 2, emerged as a compelling target for future exploration due to unanswered questions regarding the composition and structure of its atmosphere, as well as its ring system and diverse icy moon population (which includes four possible ocean worlds). Titan, as the only moon to harbor a dense atmosphere, presents some of the most complex and unique organics found in the solar system. Central to the production of these organics are chemical processes driven by low-energy photons and electrons (<50 eV), which initiate reaction pathways leading to the formation of organic species and gas phase precursors to high-molecular-weight compounds, including aerosols. These aerosols, in turn, remain susceptible to further processing by low-energy UV radiation as they are transported from the upper atmosphere to the lower stratosphere and troposphere where condensation occurs. In this review, I aim to summarize the current understanding of low-energy (<50 eV) photon- and electron-induced chemistry, drawing on decades of insights from studies of Titan, with the objective of evaluating the relevance and extent of these processes on Uranus in anticipation of future observational and in situ exploration.

## 1. Introduction

In the four decades since Voyager 1 and Voyager 2 swept past all four gas giants, modelers, astronomers, and experimentalists have been incentivized to come one step closer to examining the outer planets and, ultimately, Uranus and Neptune. While the Jovian and Kronian systems have been receiving increased scrutiny, Uranus and Neptune remain the final frontiers of planetary exploration in our solar system. Recognized as a flagship mission top priority for the next decade, the selection of the Uranus Orbiter Probe (UOP) by the *Decadal Strategy for Planetary Science and Astrobiology 2023–2032* [1] illustrates a renewed interest in the next H_2_-dominated planet. This window offers an opportunity to utilize decades of knowledge obtained from Jupiter to Saturn and the moons of these planets, as well as instrument development which could prove useful to characterize the atmospheres of Uranus or Neptune. As defined in [1], UOP would target fundamental questions about the origin and evolution of our solar system and the dynamics of chemical disequilibria across the atmosphere (Q7.1c), the chemical and physical processes influencing haze formation (Q7.3d), local ion/neutral composition (Q7.4d), and seasonal effects triggering atmospheric chemistry and variability in haze production (Q7.5c). For the latter topic, supportive laboratory and numerical studies of high- and low-pressure chemistry, reaction rates, and photochemistry will especially be relevant to improve our understanding of Uranus’ atmospheric dynamics and chemistry.

Closer than Uranus, Saturn’s largest moon Titan is the only moon in the solar system with a dense atmosphere. This atmosphere contains numerous organic molecules as well as detached haze layers resulting from intense photochemical activity in the upper atmosphere. The opaque organic haze hides a geologically rich surface made of dunes, craters, and liquid methane and ethane lakes. The Cassini-Huygens mission which arrived at Titan in 2004 (and Huygens landing on Titan in 2005), unmasked for 13 years several of Titan’s characteristics until the end-of-mission in 2017. After almost 350 years since its discovery by Christiaan Huygens in 1655, many of Titan’s secrets were about to be unveiled [2,3,4,5,6]. The unprecedented data collected by Cassini and Huygens at Titan unveiled, over a large spectrum covering the UV up to radio wavelengths, many of the atmosphere’s physical, chemical, thermal, and transport characteristics from the surface to the thermosphere. The Cassini-Huygens mission has deepened our understanding of the role of photochemistry on haze formation, benefiting from holistic experimental, modeling, and observational studies [6,7,8,9,10]. Investigations have shed light on the seasonal variations in temperature and cloud coverage, the structure of the detached haze layer, and the role of neutral-ion reactivity on the nucleation and growth of high-altitude aerosols. Still, many open questions prevail [9] and in addition to newer observations conducted to expand our knowledge of Titan’s chemical inventory, Cassini’s data heritage remains vast enough to probe for many years to come [9,11,12].

Across the four gas giant planets’ H_2_-dominated atmospheres, photochemical process are mainly driven by ultraviolet (UV) photons, magnetospheric energetic electrons and auroral deposition, galactic cosmic rays (GCR), and lightning-induced chemistry. The chemical inventory in these atmospheres directly depends on the source and intensity of photon deposition, and the photon penetration depth (see sections hereafter). In this context, the focus of this review will be on studies that have explored photochemical processes within three wavelength regions spanning UV photon energies from 3.1 to 50 eV (Table 1); this is a photon region at the source of fundamental dissociative and ionizing processes, resulting in the molecular and atmospheric diversity observed on Titan and Uranus. Region 1 contains Far-Ultraviolet (FUV) photons, Region 2 corresponds to Lyman-α radiation, and Region 3 contains more energetic Extreme-Ultraviolet (EUV) photons. Studies investigating processes induced by low-energy (<50 eV) chemistry will be reviewed according to the wavelength range as classified in Table 1.

Although the atmospheric compositions of both Titan and Uranus are distinct, decades of Titan research might help in identifying areas and techniques for both bodies needing further scrutiny into their atmospheric photochemical processes. Furthermore, the second most abundant category of exoplanets in the most comprehensive catalogues consists of mini-Neptune and Neptune exoplanets. These exoplanets have sizes of $2<R_{⊕}<6$, but the definition of this size classification itself relies on planets (i.e., Uranus and Neptune) whose characteristics themselves are poorly known. As such, further exploring Uranus (and Neptune) will not only help in exoplanetary characterization, but also in our understanding of planetary formation and the solar system’s evolution. Within our solar system, all four hydrogen-helium-rich planets hold most of the planetary mass of the solar system. Both ice giants hold a combined total of 41 moons. Our understanding of the formation and evolution of these systems relies on planetary formation and hydrodynamic models [13,14].

This review is structured into four sections as follow:

- Section 2 will include a review of the atmospheric and radiative environments of the ice giants with an emphasis on Uranus and how they differ from Titan in the Saturnian system. In addition, I will survey the current state-of-the-art body of knowledge of their chemical inventories.
- Section 3 will include a review of the state of knowledge of low-energy (<50 eV) photochemistry-induced mechanisms on Titan and Uranus, covering observations and in situ measurements, experimental simulations of gas phase and condensed state chemistry, photochemical modeling, dicationic chemistry, and recent advances in quantum chemical calculations. Important aspects of branching ratio determination will also be discussed.
- Section 4 will be dedicated to negative ions. Discoveries pertaining to negative ion chemistry on Titan deserve their own section, as their participation in molecular and haze growth requiring photochemical and radiative processes has proven to be substantial.
- Section 5 will conclude with a summary of potential future investigations needed to probe Uranus and prepare for upcoming studies before future missions to the gas giants.

## 2. The Chemical and Radiative Environments of Titan and the Ice Giants

### 2.1. Ice Giants: General Considerations

**Figure 1:**
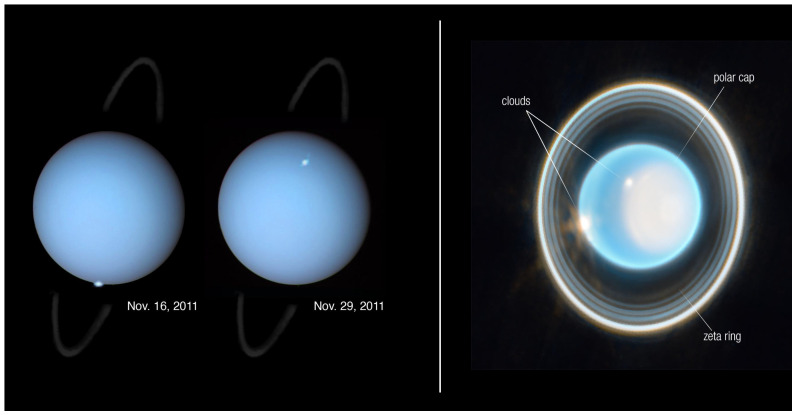
**Left**: Composite image of Uranus combining auroral observations as seen in FUV by HTS/STIS for the first time in November 2011 [22], with the planet’s blue disk seen by Voyager 2 in 1986. The faint ring system observed by the Gemini Observatory observed in 2011 is also overlaid. **Right**: JWST/NIRCam image taken on February 6, 2023. Bright sun-facing polar cap and multiple bright cloud systems can be seen. The planet’s complex 13-ring system was also captured by NIRCam with the innermost and optically thin ζ ring being visible. Image credits: NASA, ESA, and L. Lamy (Observatory of Paris, CNRS, CNES); NASA, ESA, CSA, STScI, Joseph DePasquale (STScI).

Uranus and Neptune (the “ice giants”) are the least explored planets of our solar system. To date, Voyager 2 is the only mission to have flown by anywhere close to Uranus and Neptune, at a distance of 4 $R_{♅}$ and 1/5$R_{♆}$, respectively. For the first time, these missions revealed unprecedented imagery and data of not just the two farthest gaseous planets, but also 16 new moons and 2 new rings (Figure 1). Since Voyager 2 departed Neptune’s vicinity in 1989, no other dedicated spacecraft has gone back. The ice giants stand apart from Jupiter and Saturn in their own category. On a compositional level, Uranus and Neptune both contain multiple suspected cloud layers stratified in CH_4_, H_2_S, NH_4_SH, H_2_O, and NH_3_. The temperature profiles in these planets are poorly constrained and usually assumed to follow a standard moist adiabatic and well-defined profile [15]. Radio occultation and mid-infrared measurements supported by global-average thermochemical equilibrium modeling helped in characterizing the atmospheric regions of these planets [16,17,18,19,20,21].

**Figure 2:**
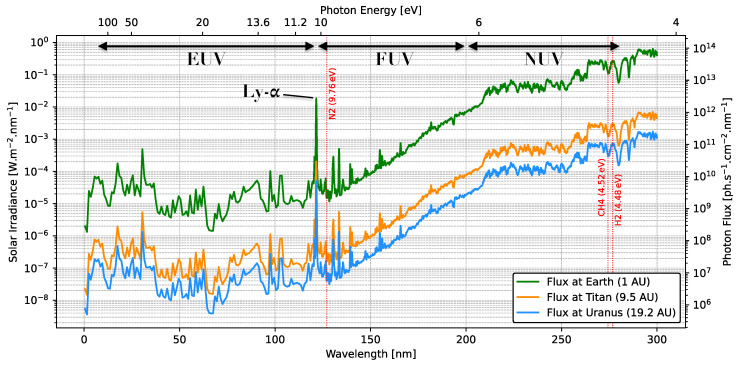
Disk-integrated high-resolution solar spectrum irradiance measured by SOLAR-HRS at Earth (1 AU) in green shown between 0.5 nm and 300 nm (data obtained with permission from [42]). The data, measured in 2022, represents a reference of a solar minimum spectrum with a spectral resolution < 1.0 nm. The spectrum is also scaled at Titan (9.5 AU) in orange and Uranus (19.2 AU) in blue here for comparison, decreasing at a scale of $1/r^{2}$. The intense Ly-α band can be seen at 121.6 nm, and red dotted lines correspond to the dissociative energy thresholds for N_2_, CH_4_, and H_2_.

A deep troposphere resides below the 0.1 bar pressure level, where temperatures are predicted to range from ∼200 K at the 10 bar level down to 50–60 K at 0.1 bar. Marking the tropopause at 0.1 bar (50 km) in both planets, the temperature inversion sets the boundary between the troposphere and the stratosphere wherein temperatures substantially increase with increasing altitude up to the thermospheric boundary located at the 1 μbar level (550 km). At these altitudes, Uranus is already much warmer than Neptune (350 K vs. 180 K). In the upper atmosphere extending up to 6000 km in the case of Uranus, Voyager 2 measurements taken by the Ultraviolet Spectrometer (UVS) show temperatures reaching 750 K near the exobase [23]. The Neptunian exobase is located around 4000 km [24]. Notwithstanding large distances from the Sun, both upper atmospheres are hotter than Saturn’s upper atmosphere, while Neptune’s is almost as hot as Jupiter’s, when considering solar heating alone [23]. Interestingly, Uranus and Neptune have both undergone an important cooling phase of their upper atmospheres since Voyager’s flybys [23,25]. Energetics and dynamics mechanisms (high-energy particle ionization, auroral processes, photoionization, solar and seasonal cycles) have been investigated to understand the intense cooling of Uranus’s atmosphere but have fallen short of combining all the complex seasonal, photochemical, orbital, and dynamical processes shaping into one cohesive theory ([23], *and references therein*). While both planets share similar temperature and atmospheric profiles, they also exhibit distinct additional characteristics. Vertical mixing is, in the standard models, assumed to be negligible at Uranus, while much more vigorous mixing is present at Neptune [15,20,26]. Eddy diffusion (**$K_{z}$**) has been described as “sluggish” by [27] with equatorial values at the homopause ranging from 3000 to 10,000 ${\mathrm{cm}}^{2}$ $s^{-1}$ [17,18,27,28,29]. Earlier modeling had placed vertical mixing even lower <100 mbar at 200 ${\mathrm{cm}}^{2}$ $s^{-1}$. Eddy diffusion is the main transport mechanism for vertical mixing but its coefficient is an important free parameter in 1D photochemical models. However, the assumption of a temperature profile which follows a moist adiabatic lapse rate does not fully reproduce the likelihood that latitudinal variations due to meridional circulation, super-adiabatic conditions, and turbulent updrafts/downdrafts may strongly influence the distribution of photochemically produced species in the atmosphere [15,21]. Coupled to auroral processes and auroral driven Joule heating, and EUV photoionization, the unique orbital geometry of Uranus (i.e., an extreme axial tilt of 97.8°) makes it a unique planet in the solar system. Although far from the Sun, solar spectrum irradiance (SSI) may reach ∼$10^{-5}$–$10^{-4}$ W $m^{-2}$ ${\mathrm{nm}}^{-1}$ at Lyman-alpha (Ly-α) wavelengths at the top of Uranus’s atmosphere (Figure 2). In addition, Uranus does not have an internal heating source, unlike Neptune [30], and the lack of understanding in the radiant energy budget of Uranus has made elucidating the planet’s weather and dynamical patterns difficult. However, recent modeling studies spanning the entire orbital period of Uranus have shed light on its internal heat flux and shown that Uranus does, in fact, possess a relatively significant internal heat source. This internal heat-to-absorbed solar power ratio is still much lower than that of the other gas giants [31]. More future studies are needed to resolve these discrepancies between models and observations [23].

### 2.2. The Atmosphere of Uranus

The atmosphere of Uranus has a mean molecular weight of around 2.3 which increases to 3.1 deeper in the troposphere below 1 bar where CH_4_ reaches a near-constant mixing ratio of ∼3% [15,19,32]. The intensity of solar EUV across the solar system decreases as the square of solar distance, where we find a solar constant of ∼14.8 W $m^{-2}$ at the Saturnian system and 3.7 W $m^{-2}$ at Uranus (Table 2). EUV intensity in the 90–110 nm range (11.27–13.78 eV, Region 3, Table 1) falls at 0.21 kR and 0.05 kR, respectively. Presented differently, Voyager 2 UVS measurements of FUV reflectance at the Uranian subsolar point (i.e., close to the rotation axis) by [33] helped constrain the CH_4_ column abundance even before the closest approach. At the same time, occultation experiments helped quantify the column abundances of H_2_, H, and C_2_H_2_ [34]. These occultation measurements found that the opacity could be explained by the dominating presence of Rayleigh scattering of H_2_ along with the presence of a Raman line at 1280 Å and C_2_H_2_ absorption near 1300 Å and 1500 Å [27,33]. More recently, Ref. [22] conducted the first Earth-based detection of an auroral event (transient emissions on the order of 1–2 kR) at Uranus, observed in the FUV in November 2011 during an intense solar wind event. These observations clearly indicated that there exist variations in the configuration between the solar wind and the magnetosphere, and therefore the thermal and compositional structure of the upper atmosphere. Later measurements by the Hubble Space Telescope (HST) permitted a re-analysis of Voyager 2’s albedo measurements from [35], which brought down its value from ∼20–30% to 5–10% between 135 and 155 nm (8.0–9.19 eV, Region 1, Table 1) [36]. The emission features studied therein would have required an estimated energy flux of the precipitating electrons of 0.04–0.07 erg ${\mathrm{cm}}^{-2}$ $s^{-1}$, but sensitivity shortcomings prevented any determination of their actual energy in the 20 eV–20 keV range. Nonetheless, this first Earth-based detection of a transient auroral H_2_ emission came in good agreement with the Voyager 2-era disk average flux upper limit of 0.008 erg ${\mathrm{cm}}^{-2}$ $s^{-1}$ by [37]. The modeled H_2_ spectrum resulting from solar photons and precipitating electrons considered low-energy (centered at 20 eV) and higher energy (3 keV) electrons. The calculated $\chi^{2}$ dependence of the precipitating flux showed little difference between these two energies and that one single transient event would have caused a total electron flux enhancement by a factor of 4–9 [36]. Overall, both Voyager 2 and Earth-based observations have contributed to a better understanding of auroral and photochemical events in the upper atmosphere of Uranus. However, we are still far from an accurate global picture involving complex latitudinal, temperature profile variations, hydrocarbon contributions, magnetospheric configuration, and accurate knowledge of solar FUV/EUV fluxes [38]. As such, a more accurate description of these fluxes (extrapolating also into more energetic X-ray fluxes) will ultimately help in better characterizing exoplanet environments and their atmospheric composition [38,39,40,41].

The first recorded molecular detection in Uranus occurred in 1869 by [43]: *“Dans le vert et dans le bleu il y a deux raies très-larges et très-noires”*. Unbeknownst to the astronomer and priest that this detection was in fact that of molecular hydrogen, this was followed by observations of unresolved faint bands by [44]. Then, *“The remarkable absorption taking place at Uranus shows itself in six strong lines”* [44,45]. This first set of two observations acquired very faint bands, but it was not until 1934 that the first observations firmly detected CH_4_ as being the first confirmed compound in the atmosphere [46]. Eighteen years later, the H_2_ ro-vibrational band at 8270 Å was discovered [47], and nearly thirty-four years later, accurate measurements of the H_2_:He mole fraction were conducted and trace species were discovered by the International Ultraviolet Explorer [48] and Voyager 2 [49]. At present, 9 neutral molecules have been directly detected in the atmosphere of Uranus (see Table 3 for an elemental composition comparison between Uranus and Titan).

**Figure 3:**
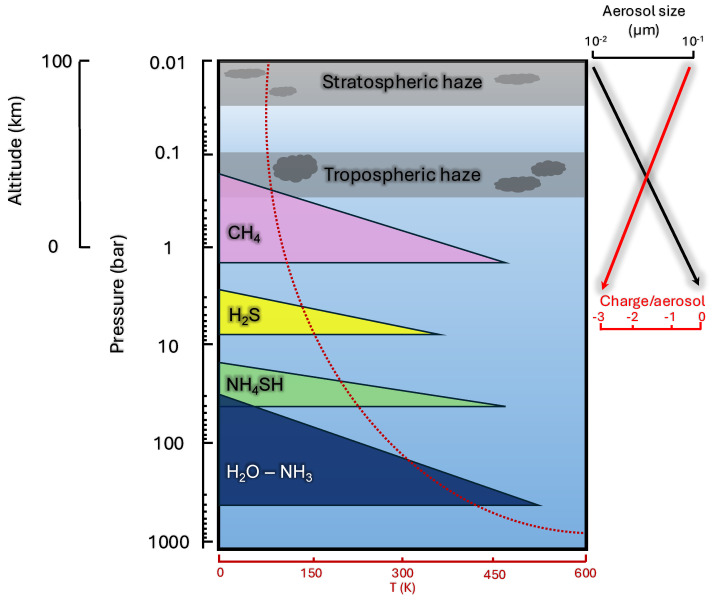
Standard picture schematic of the atmospheric profile of Uranus with the tropospheric and stratospheric haze layers, the CH_4_, H_2_S, NH_4_SH, and H_2_O−NH_3_ cloud decks, and the approximate temperature profile [15,18,19,20,60,61]. The general, approximate trends across the haze layers for aerosol particle size distribution (in μm), and the distribution of the mean charge per aerosol from [62] at altitudes of 0.01–3 bar are also given.

The atmosphere of Uranus starts with a deep troposphere which rises up to 1 bar, and whose composition and structure are still, to a large extent, poorly known [15,21]. CH_4_ is in abundance and drives the moist convection in the troposphere, before it condenses due to the cold temperatures near the tropopause (1–1.5 bar). In models assuming a temperature profile following a moist adiabat, all expected CH_4_, H_2_S, NH_4_SH, and H_2_O−NH_3_ species form stratified, well-defined cloud decks below 1 bar (Figure 3). Methane, like all other species, display latitudinal and temporal variations and as a result are important indications of underlying seasonal and/or vertical transport [18,21,55]. Indeed, as explained in [21], tropospheric updraft of methane and other hydrocarbons is unlikely given the cold trap at ∼50 K. However, mechanisms such as mid-latitude tropospheric updraft could in theory transport CH_4_-rich air mass up into the stratosphere. Or, adiabatic warming may occur, thus moving subsiding air from the stratosphere into the troposphere [18,56] (a similar seasonally driven stratospheric circulation was seen on Titan, discussed later). Modeling of the visible/near-infrared reflectivity of the haze profile on Uranus includes several uncertainties but was recently studied by [57]. Their conclusion was that the atmosphere consists of three “detached” haze layers. (1) A vertically extended photochemical haze layer $<10^{-2}$ bar which is produced in the lower to mid stratosphere. Haze particle are then relatively mixed to lower altitudes by Eddy diffusion. (2) Right above the CH_4_ condensation altitude at ∼1 bar, haze particles of moderate size (∼1 μm) constitute a thin but highly opaque cloud structure composed of an important mixture of CH_4_ ice and photochemically produced particles, absorbent of UV and long-wavelength photons. It is at this level that ice/haze particles are expected to act as cloud condensation nuclei (CCN). (3) Finally, a deeper and darker aerosol layer for *P* > 5–7 bar would be consistent with the presence of an H_2_S-based layer mixed with photochemically generated aerosols and other ices. The modeled haze particles are predicted to scatter light at 500 nm while absorbing photons at longer wavelengths. Future experimental and numerical studies of the optical and physico-chemical properties of these particles would help in constraining the vertical structure of Uranus’ atmosphere which remains largely uncertain. Support from telescopic observations is ongoing [58] and recent surveys using the Lowell Observatory and HST have highlighted the dependency of cloud brightness variations with CH_4_ distributions and orbital position [59].

A number of species such as HCN, PH_3_, GeH_4_, HCl, and CH_3_SH expected to be in disequilibrium have been speculated from thermochemical modeling to survive in the upper troposphere, thus importantly bridging the deep troposphere composition with the stratosphere [20]. There are however many uncertainties surrounding this chemistry such as: knowledge of the deep tropospheric temperature profile, ice nucleation at low temperature, kilobar-level and high-temperature reactions rates, diffusion effects, and non-ideal gas behavior, all of which are factors of uncertainty that have been highlighted in [20]. Above the 1 bar pressure (Figure 3), the stratosphere extends up to ∼1 μbar where temperatures rapidly reach 300 K, following the moist adiabat, and finally reach 750 K at the exobase [18]. This vertical structure interpretation is almost certainly erroneous for the reasons mentioned previously, and the reality is likely to involve many altitudinal, latitudinal, and seasonal variations [15]. These atmospheric parameters will regulate the distribution of species in the atmosphere, and these species will also influence the cloud structure and haze formation of the planet. Therefore, it is crucial to obtain a better understanding of the chemical inventory and physico-chemical processes in Uranus.

### 2.3. Chemical Inventory in Uranus

This section will focus on the available inventory of detected species in the atmospheric column of Uranus. The origin and photolytical evolution of the species are still largely unknown, and as pointed out in [63] the differences observed with the other gas giants may result from different production/loss photolytic rates, variations in atmospheric mixing, extent of ion-neutral chemistry, auroral conditions, and exogenic material influx. To date, stratospheric column abundances have been measured for a total of 9 neutral molecules and one isotopic D/H (measured in H_2_) with a value of $4.4\times 10^{-5}$ (Table 4). They are composed of alkanes (CH_4_, C_2_H_6_), alkenes (C_2_H_4_), alkynes (C_2_H_2_, C_3_H_4_, C_4_H_2_), and oxygenated molecules (CO, CO_2_, and H_2_O). Since no dedicated mission has yet flown to Uranus, new molecular discoveries happen at an irregular pace with long-term windows that depend on telescopic observations [64]. However, the benefits of a descending probe into the Uranian atmosphere are undeniable and would increase our understanding of the chemical, radiative, and optical properties of the atmosphere [63,64]. Such an in situ study would help us constrain the gas and haze composition with the energy deposition distribution in the atmosphere.

Multiple photochemical models have been developed, often studied in tandem with Neptune, to understand the global-averaged hydrocarbon distribution on both planets [17,78,79,80,81,82,83,84]. Generally, they did not however include seasonal and latitude effects on the stratospheric abundance of hydrocarbons. Ref. [18] provided the first one-dimensional (1D) model to incorporate such effects and to track the time- and location-variable influencing the distribution of stratospheric species. This study showed Neptune to have very similar seasonal dynamics to Saturn, while Uranus’s were found to be different, primarily due to its $97.8^{{}^{\circ}}$ axial tilt and weak vertical transport. The main hydrocarbons (Table 4) are confined at low altitudes and concentrated at high polar latitudes. The time constants and photochemical lifetimes of these species (except for C_2_H_6_) were found to be larger than their loss rates, furthering the stratification of hydrocarbons [18]. More recently, a 1D seasonal radiative-convective equilibrium model developed by the Laboratoire de Météorologie Dynamique historically used for Jupiter, Saturn, and exoplanets investigated the origins and evolution of the thermal structure of Uranus and Neptune [85]. This study stressed the importance of knowing the optical properties of haze particles, and changing optical indices will alter the warming or cooling rates in the atmosphere. In addition, a precise CH_4_ abundance constraint, with or without haze, will significantly impact the retrieved temperature profiles. Thus, discrepancies between observations and the simulated thermal structure is likely to hint at variations in the latitudinal stratospheric distribution of CH_4_ and haze particles [85]. A better characterization of these variables will be crucial to understand the extent of Uranus’s thermal impact on the atmospheric composition. A re-analysis of Uranus’s energy budget has recently challenged the long-held assumption of a lack of internal heat source [31].

### 2.4. The Atmosphere of Titan

**Figure 4:**
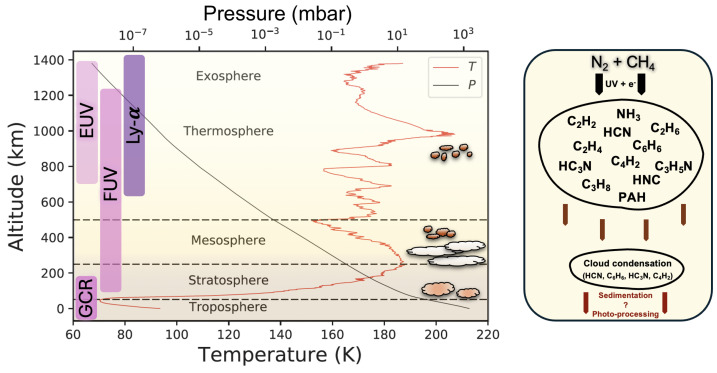
Vertical temperature (red) and pressure (black) measurements conducted by Huygens during its descent down to the surface of Titan. Data obtained from the Planetary Data System: Planetary Atmospheres Node. Adapted from [92].

Titan, the largest moon of Saturn, is uniquely recognized as the sole known natural satellite to possess a dense atmosphere predominantly composed of molecular nitrogen N_2_ (∼98%) and methane, CH_4_ (∼2%). This reducing atmosphere is stratified into five primary layers—namely, the troposphere, stratosphere, mesosphere, thermosphere, and exosphere (Figure 4)—each exhibiting planetary-scale dynamical, thermal, chemical, and seasonal variations. Temperatures are the coldest (∼70 K) at the tropopause. The formation of the photochemical haze that envelopes Titan is regulated by gas-phase molecular precursors, which are generated in the upper atmosphere through high-altitude N_2_ and CH_4_ photolysis and radiolysis processes. These precursors consist of hydrocarbon radicals (e.g., CH_2_, CH_3_), in addition to more complex hydrocarbons, nitriles, and potentially polycyclic aromatic hydrocarbons [86]. Energetic sources facilitating these high-altitude chemical reactions include solar UV photons, solar X-rays, galactic cosmic rays, Saturn’s magnetospheric energetic electrons, and the solar wind [87,88]. The Cassini-Huygens mission, over a duration spanning 13 years, conducted an extensive study of Titan, taking direct measurements of the composition in neutrals and ions within Titan’s upper atmosphere [86,89]. These investigations elucidated the complexity of Titan’s upper atmospheric chemistry, characterized by radicals, neutrals, positive and negative ions, as well as the early stages of solid haze particle formation. On numerous occasions, the measured abundances of specific transient ions such as CH_4_^+^, HCNH^+^, C_2_H_5_^+^ exceeded predictions from prior models (e.g., [90]). Conversely, the concentrations of certain heavy hydrocarbons and nitrogen-bearing neutral molecules, as determined by the Ion and Neutral Mass Spectrometer (INMS), were found to be lower than anticipated by ion-neutral models ([91], *and references therein*). These findings emphasized the crucial role of magnetospheric electron precipitation and diurnal solar energy variations in modulating molecular distributions within the upper atmosphere.

Connecting Titan’s upper atmosphere dynamics with incoming solar and magnetospheric energy deposition started long before Cassini’s arrival at Saturn. Benefiting from earlier Voyager 1’s flyby of Titan in 1980 [93] at a time when Titan was located inside of Saturn’s magnetosphere, it became apparent that Titan was at that time in a moon-solar wind configuration akin to Venus [94]. Well over a decade later, refinements stemming from occultation and plasma experiment measurements led to the expansion of multiple photochemical models [95,96,97,98,99,100] along with models incorporating thermal and suprathermal electrons calculations [99]. Based on these measurements, it was found that N_2_ EUV airglow emission initiated by photoelectron impact dominated over N_2_ airglow triggered by magnetospheric electron impact [99]. Titan’s ionosphere operates as a photochemical factory at the interface between incoming solar photon and magnetospheric electron fluxes, and the underlying first steps of molecular and aerosol growth [101].

The solar flux reaching Titan is around 14.8 W $m^{-2}$ (Table 2) and the photon flux at Titan ranges from ∼$10^{6}$ ph $s^{-1}$ ${\mathrm{cm}}^{-2}$ ${\mathrm{nm}}^{-1}$ (@60 nm) to $10^{7}$ ph $s^{-1}$ ${\mathrm{cm}}^{-2}$ ${\mathrm{nm}}^{-1}$ (@110 nm) ([102] and Figure 2). As the primary carrier of VUV energy across the solar system (Region 2, Table 1), Ly-α (121.6 nm) has the capacity to penetrate into Titan’s upper atmosphere where haze particle formation is initiated. VUV radiation reaches a factor of 100 higher in intensity at 121.6 nm than the baseline at 60–110 nm [102]. Longer wavelength UV photons may even reach lower stratospheric levels which continuously exposes the growing aerosols to the UV radiation ([103] and Figure 4). Modeling of photon and photoelectron deposition on Titan by [103] compared with Cassini Plasma Spectrometer Electron Spectrometer (CAPS/ELS) electron flux measurements at 1014 km showed high matching with the observations. Their comparison confirmed the predominant activating role of photon and photoelectron contribution on the dayside compared to the nightside fluxes derived from the contribution of magnetospheric electrons. The peak photon contribution lies around the ionospheric peak ∼1000 km whereas secondary electrons produced from X-rays contribute mostly at lower altitudes (700–900 km). At the latter altitudes and below the tropopause, GRC radiation is estimated to induce ∼10 and ∼0.1 ionization events ${\mathrm{cm}}^{-3}$ $s^{-1}$, respectively [104]. At higher altitude, it is estimated that Ly-α photons account for about 75% of all photo-dissociated CH_4_ [10,105].

Deeper in the atmosphere, shaped by seasonal dynamics, large polar cloud systems have been observed for over 30 years in Titan’s stratosphere. Subject to meridional and vertical transport, these stratospheric clouds have been particularly well-studied in polar winter conditions where they contain several ice spectral signatures (see Section 3) seen in the mid- and far-infrared [106]. Their formation occur at higher altitudes (>168 km) than predicted by models [107] and it is likely that they contain ice co-condensates given their overlapping spectral features [108,109]. An important HCN-rich cloud system was even observed at z=300 km in the south pole after a substantial cooling event in the polar vortex [110,111]. As a result, climate and circulation models have had to be refined and updated after such observations where they could not account for (i) rapid and dramatic changes in the seasonally defined temperature variations, and (ii) shortcomings due to lack of theoretical and experimental aerosol microphysics data [8,111,112,113,114,115,116]. While these discoveries have helped constrain parameters in General Circulation Models (GCM), they have also left many unanswered questions regarding their ice composition and potential for photochemical evolution [104,108,117,118,119,120,121]. While the intermediate chemical and physical pathways connecting the formation of aerosols and clouds with the gas phase precursors are still not fully resolved, Titan’s chemical catalogue remains one of the most complex in the outer solar system.

### 2.5. Chemical Inventory in Titan

According to the most recent photochemical models, Titan’s atmosphere is composed of: hydrogen and hydrocarbons, nitrogenated compounds, oxygenated molecules, and presumed but undetected polycyclic aromatic hydrocarbons (PAH) and reduced phosphorous and sulfur compounds [70,122,123,124,125,126,127] (See Table 3 and Table 4 for summaries of molecules detected, and [10] for an extensive review of Titan’s atmospheric composition). The ionization rate peaks around 1150 km. Employing Chapman layer theory, ref. [98] calculated the peak electron density to occur at 1200 km, [94] at 1175 km, and [128] at 1040 km. The latter study anticipated HCNH^+^ to be the dominant ion, utilizing a model comprising over 60 species and 600 reactions. Subsequently, Ref. [100] enhanced this analysis, pinpointing the predominant *m/z* 28 peak as HCNH^+^. Chemical models have helped explain the gas phase chemistry involved in the production of Titan’s aerosols (e.g., [70]). Alongside those models, laboratory experiments have helped in vetting the reaction networks through the study of specific channels involving neutral and charged molecular species. Furthermore, photochemical and microphysical models have investigated the dusty nature of Titan’s ionosphere (>900 km in the atmosphere) and characterized the interaction between the aerosols and charged particles [129]. More specifically, by using a wide array of energy sources and coupling extensive lists of ion-neutral reactions, models have given insights into the first steps linking small hydrocarbons with larger molecules [70,126,127,130]. Ion-molecule reactions are thought to be directly relevant to aerosol growth, and are controlled by the relative abundances of the two initial neutral main constituents, N_2_ and CH_4_ [129]. The dissociation of CH_4_ by EUV photons results in the formation of methylene ^1^CH_2_ in its excited state with a dissociation yield of 0.48 for Ly-α photons, and 0.50 in the FUV (Reaction (1)),

(1) CH4+hν⟶CH21+H2

More energetic photons in the EUV (<121.6 nm, Table 1) will preferentially dissociate methane to form the methylene radical ^3^CH_2_ in its ground-state (Reaction (2)), while Lyman-α radiation will also form the methyl radical CH_3_ with a dissociation yield of 0.42 (Reaction (3), [10]).

(2) CH4+hν⟶CH23+2H

(3) $${\mathrm{CH}}_{4}+h\nu ⟶{\mathrm{CH}}_{3}+H$$

**Figure 5:**
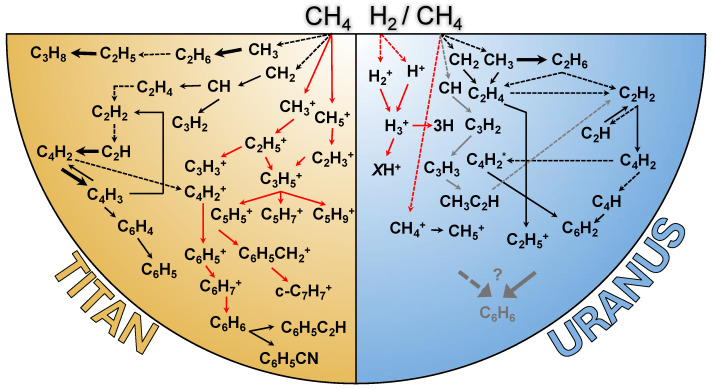
Simplified schematic diagram showing the predominant neutral gas phase hydrocarbon formation pathways in the upper atmospheres of Titan and Uranus, based on available combined observations from Cassini, Voyager 2, Spitzer, IRTF, Hubble, and photochemical models [10,17,21,23,27,34,49,52,70,78,79,95,126,132,133,134,135,136,137,138,139,140,141,142,143,144,145]. Photolysis reactions are shown with dotted arrows, termolecular reactions in thick arrows, and all other types of reactions (neutral, proton abstraction, radical-radical, and radical-molecule reactions) are shown in thin black arrows. Reactions involving ions are shown in red. Note that the reactions in gray leading to the formation of methylacetylene (CH_3_C_2_H) on Uranus are derived from the photochemical model by [140] developed for the Jovian atmosphere, akin to formation pathways proposed by [146] to explain the as-of-yet undetected C_6_H_6_ on Neptune.

The formation of these first radicals in Titan’s upper atmosphere by FUV, Ly-α, and EUV photons is a crucial starting point towards the formation of the first intermediate, and then the larger hydrocarbons. Thence begins a long series of cascade of dissociative recombination, proton abstraction, and polymerization reactions that will ultimately lead to the formation of photochemical aerosols (see [70] and Figure 5). The absorption of low-energy (<10 eV) photons as well as much higher energy EUV, by radicals is still poorly understood. Long sought for in the Titan community, CH_3_ has perplexed photochemical modeling studies for many years due to its non-direct detection. First, because CH_3_ photolysis branching ratios are not well known. Second, CH_3_ can be an important source leading to the formation of ^1^CH_2_. Third, CH_3_ production actually expedites CH_4_ loss in Titan’s atmosphere, where up to 65% of CH_3_ results from CH_4_ photolysis with a predicted mole fraction of $10^{-4}$ at 1000 km, the highest abundance of any of the small radicals in the atmosphere [70]. As a result of this complex radical photochemistry, two hydrocarbons are then produced early on (Figure 5). First, ethylene C_2_H_4_ forms through the radical-molecule Reaction (4) and then ethane C_2_H_6_ through the radical-radical Reaction (5) at higher pressure where three-body reactions are permitted, with a peak production at 800 and 200 km [70].

(4) $$\mathrm{CH}+{\mathrm{CH}}_{4}⟶C_{2}H_{4}+H$$

(5) $${\mathrm{CH}}_{3}+{\mathrm{CH}}_{3}+M⟶C_{2}H_{6}+M^{★}$$

At these altitudes, FUV photons continue to interact with hydrocarbons such as C_2_H_2_ with low-energy (between 5.5 eV and 12.4 eV) photons which will result in the production of C_2_ and C_2_H, two radicals involved in significant methane-depletion and hydrocarbon-formation mechanisms [10]. These reactions underscore the contribution of CH_4_ photo-degradation by low-energy (<20 eV and especially Ly-α) photons and the important role radicals play towards the growth of hydrocarbons in a relatively methane-rich environment. This role is particularly important since it also leads to the very molecules that will condense into the lower-altitude clouds and regulate the radiative dynamics of the stratosphere. In recent observations of the late northern summer, CH_3_ was detected in the mid-IR by JWST which showed excellent agreement with photochemical modeling abundance results [131], confirming its important role in the neutral atmosphere.

### 2.6. From Dynamics to Haze Stratification and Evolution

As outlined above (Section 2.1), atmospheric dynamics play a central role in the transport mechanisms influencing photochemical products, haze formation, and their evolution over time. As such, comparative planetology among all jovian planets and Titan seems necessary to better constrain the underlying mechanisms at play in these cold atmospheres. Irrespective of the planet, constraining each planet’s temperature profile is fundamental to derive other atmospheric properties. For example, the refractivity, convection and wind parameters, chemical equilibrium, abundance and condensation of trace gases, cloud formation, precipitation, and particle size distribution are all processes dependent upon or linked to the local temperature gradient. The evolution of haze layers (and the condensation levels of precursor trace gases) is of particular interest since its distribution is shaped by the adiabatic regime and temperature profile [147,148]. With little data regarding this regime and temperature profile on Uranus, it is thus hard to infer any detailed description of the CH_4_ (and other condensates) cloud deck stratification and evolution. Moreover, storms and seasonal variations are also likely to affect downdraft and updraft transport, thus modifying a well-stratified cloud condensation structure [148]. Conversely, a poorly mixed stratosphere on Uranus may expose certain haze strata to long-timescale radiative influx, thus potentially affecting their composition. Alternatively, subsiding volatile-rich air masses resulting from circulation cell reversal on Titan enabled the formation of cloud condensation at higher altitude than expected [111,115]. In parallel, such transport also allows multiple species (C_6_H_6_, C_4_H_2_, HCN, etc.) to co-condense, resulting in more complex photochemistry than previously thought. Additionally, a recent re-analysis of solar occultation of Titan prior to the northern spring equinox revealed “leaking” CH_4_ from the troposphere into the stratosphere, which reinforces the need for better characterization of the thermal fluxes involved [149]. Furthermore, free parameters such as $K_{z}$ are poorly constrained and in Titan’s stratosphere, $K_{z}$ values used in photochemical models can vary up to a factor of 100 [126,150]. In summary, both Titan and Uranus provide a unique H_2_-rich vs. N_2_-rich portrait to investigate their intrinsic dynamical, thermal, and chemical behaviors. Ultimately, improved knowledge of the local temperature and compositional conditions where hazes and clouds form can help guide future theoretical and experimental studies that aim to reproduce the compositional and radiative conditions of Uranus.

## 3. Photochemistry vs. Radiative Chemistry: Competing Processes and Role in Haze Formation

### 3.1. Fundamental Processes

Photochemical reactions are fundamental to the plurality of chemical inventories in space since they pertain to electronically excited states and are governed by the law of reciprocity (Bunsen-Roscoe law) in that their reactivity is proportional to the fluence, regardless of exposure time. About nine different types of photochemical reactions relevant to planetary and interstellar conditions exist, and almost twice as many ionizing radiation-based reactions [151,152,153]. Among these, six of the fundamental reactions are listed hereafter:

- photodissociation: A*⟶B+C
- quenching: A*+B⟶A+B
- luminescence: A*⟶A+hν
- photoisomerization: A*⟶B
- bimolecular reaction: A*+B⟶C+D
- hydrogen abstraction: A*+RH⟶AH+R

These reactions occur constantly in planetary atmospheres and serve a fundamental role to initiate gas phase cascade reactions involving radicals and their excited states [10]. Photodissociation reactions primarily contribute to the destruction of CH_4_ to form the first radicals (see Section 2), while proton abstraction is thought to be involved in the loss of C_2_H_2_ to form the first carbon chain anion C_2_H^−^ [10]. Other pathways obtained by [154] applied to the interstellar medium (ISM) have also included bimolecular reactions as an important means of anion growth. Furthermore, infalling H_2_O on Titan is expected to participate in the bimolecular reaction with the excited-state N(^2^D) atom in the ionosphere [155]. Other reactions such as ion-pair formation or sensitization are also included in photochemical models but their involvement not as well understood [152]. Note that these channels are driven by photons in the NUV to FUV range. In the solar system, and in particular in the outer solar system, low-energy (<10 eV) photons may originate isotropically from (inter)stellar background radiation, particularly the interstellar radiation field (ISRF) and scattered Ly-α in the local interplanetary medium (LIPM) resulting from H_2_’s excited Lyman and Werner bands, also called the Solomon process. As seen in [18], accurate modeling of these UV sources are required to understand seasonal effects, particularly at the poles of Titan and Uranus where shadowed conditions can last up to several years in the winter hemispheres. These regions can thus be more favorable to rapid CH_4_ photolysis with its dissociation energy threshold of 4.52 eV (Figure 2). Likewise, with a low-energy dissociation threshold of 4.48 eV, H_2_, a symmetrical molecule, presents only one vibrational mode as its indirect dissociation occurs through the continuum of the ground electronic state. Notwithstanding limiting cold temperatures, heterogenous chemistry may also come into play where solid grains or polycyclic aromatic hydrocarbons (PAH) may catalyze H_2_ formation through chemisorption or physisorption, a process well-studied in photodissociation and other PAH-enriched regions [156].

In addition, radiation chemistry includes pathways pertaining to processes resulting from the interaction between the gas or condensed phases and ionizing radiation above the ionization threshold ∼10 eV. A unique feature emanating from radiation chemistry is the generation of a cascade of low-energy secondary electrons [157,158,159,160]. These electrons’s energy have been shown to induce substantial chemical changes on cosmic ice grains, and have even been proposed to participate in the formation of certain prebiotic molecules [161,162]. In [159], the authors studied radiolytic mechanisms of degraded NH_3_ ices in ISM-like conditions, bombarded with ∼1 keV electrons accompanied by low-energy electrons (∼7 eV). The authors found that non-ionizing radiation of condensed NH_3_ (ammonia expected to be the most abundant nitrogen-bearing compound in the ISM, while also present in the gas phase formed in Titan’s ionosphere, and in the condensed state in Uranus’s troposphere, Figure 3) was responsible for the formation of hydrazine (N_2_H_4_) and diazene (N_2_H_2_) in the condensed phase. It was proposed that a first step involved the dimerization of the NH_2_ radical due to >1 keV impact, followed by a favorably energetic dissociative electron attachment (DEA) at 6 and 10 eV [159]. As surveyed in [153], low-energy secondary electrons generated by radiation chemistry can lead to reaction pathways that are otherwise not relevant to photochemistry. Unlike photons, electrons can induce singlet-to-triplet transitions via exchange interactions with electrons [158]. Additionally, electrons can be transiently captured by molecules, forming temporary negative ions (TNIs) through resonant processes such as shape and core-excited resonances [163,164]. These TNIs may undergo dissociative electron attachment (DEA), leading to bond cleavage and the generation of reactive fragments capable of further chemical transformations [153,165]. Through the combination of the yield of secondary low-energy electrons generated by radiation chemistry with UV photons dissociating and exciting molecules, these competing processes contribute to diversifying the molecular inventory necessary for the rapid molecular growth leading to the formation of aerosols in planetary atmospheres. The following reaction mechanisms are only a handful [166,167].

- Ionization: $\mathrm{AB}⟶{\mathrm{AB}}^{+}+e^{-}$
- Ion dissociation: ${\mathrm{AB}}^{+}⟶A^{+}+B$
- Ion-molecule reaction: ${\mathrm{AB}}^{+}+\mathrm{BC}⟶\mathrm{Products}$
- Electron attachment: $e^{-}+M⟶\mathrm{Products}$
- Fluorescence: AB*⟶AB+hν
- Excimer formation: $A*+A⟶{A_{2}}^{*}$

Another process still poorly understood concerns multiphoton and fluorescence chemistry, which was first observed by [168], resulting from the photodissociation of simple hydrocarbons such as C_2_H_2_ and C_2_H_4_. In that study, UV photon (193 nm) absorption alone was not able to explain the fluorescence of free radical photo-products. To account for this discrepancy, the authors put forth the proposition of a sequential absorption scheme where molecule AB^*^ absorbs a secondary photon, a concept revisited in [169] to understand cometary emission of C_2_. Relevant to Uranus’s lower atmosphere, the multiphoton ionization of H_2_S [170,171,172] or even PAHs (e.g., [173]) have been studied in order to understand the ionic fragmentation patterns involved in the UV-VIS region. The processes involved in radical photochemistry and secondary photolysis still remain to be characterized in the context of planetary atmospheric chemistry (Table 5).

Pathways involving ions are too numerous to include here and the reader is referred to [10,18,70] for an updated in-depth overview of known reaction schemes for Titan and Uranus. Instead, a focus hereafter will be placed on the competition between photochemical vs. radiative processes (impacting excited and dissociative ion chemistry, respectively) since ion reactivity remains a crucial component to investigate to understand haze formation in the solar system, interplanetary, and interstellar ices [152,153,160,166]. Numerous studies have investigated the photochemical effects induced by Ly-α on interstellar matter which represents a good benchmark for heterogeneous chemistry conducive to the formation of organic molecules in the solar system [160,161,167,179,180]. Furthermore, FUV, EUV, and even NUV radiation has been shown to induce chemistry in the condensed state in laboratory Titan ice and aerosol analogues [104,119,120,121,178]. In Titan’s upper atmosphere, photochemically produced haze will indeed interact with these UV photons, thus modifying their composition and the ice CCN down to mesospheric and stratospheric altitudes. The extent of this hν-induced chemistry remains far from being fully characterized, and the task becomes even worse for Uranus. Here, we will present a summary of studies that have probed Titan’s and/or Uranus’s photochemical processes vs. those that have investigated radiation chemistry at energies <50 eV utilizing (primus) Cassini/Voyager or ground-based observations, (secundus) experimental analyses simulating Titan-like low-temperature reactivity in the condensed state initiated by low-energy (<20 eV) photons and electrons, (tertius) advances in the theoretical study of branching ratios and their relevance in the VUV, and (quartus) considerations of the relatively poorly understood role of dication species and their reactivity through vertical ionization processes.

### 3.2. Observational Considerations: From Low-Mass to Intermediate-Mass Molecules

#### 3.2.1. Low-Mass Species

**Figure 6:**
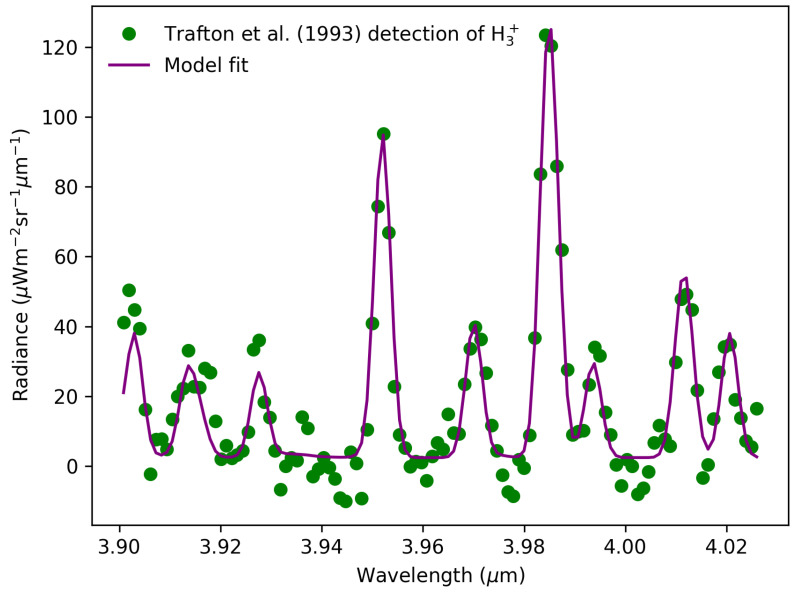
Observed spectrum of H_3_^+^ (green dots) detected by [139] between 3.89 and 4.03 μm, a range which includes the region with multiple lines of the *Q* branch of H_3_^+^, with the most intense *Q*(3) band at 3.985 μm [143]. A model fit using the h3ppy open source package computed with a rotational temp 740 K is shown in purple. Here, the model retrieves an H_3_^+^ column density of $4.42\;\times \;10^{14}$ $m^{2}$ (https://github.com/henrikmelin/h3ppy, accessed on 21 May 2025).

As part of this discussion and the species discussed below, we will follow the simplified classification from [181,182] separating low Z and high Z elements found in Jovian planets, resulting from photochemistry and radiation chemistry processes. Low Z elements revolve around H and He, while high Z elements concern any species with a mass higher than He. A focus will be placed primarily on the role of H on Titan and Uranus, and H_3_^+^ on Uranus. About 20 years separate the first detection of H_2_ on Uranus [47] and on Titan [183]. Two decades later, H_3_^+^ was detected on Uranus, serving as an important temperature proxy in the upper atmosphere from its emission intensity-dependence on solar activity [139,184]. Figure 6 shows the observed spectrum of H_3_^+^ and the intense *Q*(3) band centered at 3.985 μm with the open source model fit using the h3ppy package (version 0.6.1). The chemistry of H_3_^+^ on Uranus mainly involves daytime and nighttime processes. EUV photons (>10 eV) on the dayside of Uranus ionize H_2_ which produces H_2_^+^ and a free electron (Reaction (6)). H_2_^+^ then reacts with the background H_2_ to produce H_3_^+^ through the exothermic Reaction (7). Recently, other channels have been proposed to explain H_3_^+^ formation, requiring impact ionization of C_2_H_6_ by higher energy electrons (300 eV) [185] and from doubly ionized cyclopropane C_3_H_6_ [186]. The atomic yield of hydrogen through these channels is an important consideration since, as pointed out in [79], (i) H loss by chemistry (Reactions (8) and (9)) influences the recycling and abundance of CH_4_, and (ii) controls the production of polyacetylenes such as C_4_H_2_ from competing quenching of metastable excited C_4_H_2_^**^ by H_2_ and bimolecular reaction pathways. Note that H_3_^+^ was recently detected for the first time at Neptune with JWST [187].

(6) $$H_{2}+h\nu ⟶{H_{2}}^{+}+e^{-}$$

(7) $${H_{2}}^{+}+H_{2}⟶{H_{3}}^{+}+H$$

(8) $$H+C_{4}H_{2}+M⟶C_{4}H_{3}+M$$

(9) $$H+C_{4}H_{3}⟶C_{4}H_{2}+H_{2}$$

C_4_H_2_ being a strong absorbent of UV light, it is likely to be excited into the metastable state C_4_H_2_^**^ between 180 and 260 nm, a process more likely to occur on Uranus than in the nitrogen-rich atmosphere of Titan [79]. These reactions were later taken into account in [138], and the later detection of C_4_H_2_ by [52] raises the question of what the extent of these small hydrocarbons on Uranus is and the competing roles between photochemistry, radiation chemistry, and condensation processes which might affect the abundances of polyacetylenes in the atmosphere. Furthermore, as shown in stratospheric photochemical modeling [18], the latitudinal distribution of hydrocarbons is strongly dependent on seasonal effects (i.e., solar radiation), and thus on photochemical efficiency. On Titan, the chemistry of low Z elements is relatively less influential than that on Uranus, since H_2_ concentrations on Titan are much lower (∼0.4% [188]). Still, atomic hydrogen is thought to originate mainly from CH_4_ photolysis (other subsurface sources may also exist [189]), although its exact vertical profile and source/sink equilibrium are still debated ([10], *and references therein*).

#### 3.2.2. Higher-Mass Species

Higher Z-mass species (*m/z* > He) between Titan and Uranus share one common chemical entry point: the photo-destruction of CH_4_ (Figure 5). As discussed above, C_4_H_2_ with a mass of 50 amu remains the highest mass of a molecule detected on Uranus, while negatively charged particles with masses of over >10,000 amu are the largest detected on Titan (albeit with no exact molecular identification to date). If, on Uranus, intermediate mass molecules (C_2_H_2_, C_2_H_6_, C_4_H_2_) do condense out near their production altitude [138], UV-driven solid-state chemistry becomes a possibility, although this is at present a field almost completely unexplored. As pointed out in [142], photolytic destruction branching ratios of C_2_H_2_ and formation kinetics of C_2_H_6_ from ^1^CH_2_ are also not well constrained and model-sensitive, which can result in significant modeled abundance variations. The formation of PAHs is also an unresolved question, as they have only been indirectly identified on Titan [190], while C_6_H_6_ alone has so far not been detected on Uranus. Recent experimental work in this field has shown promising results (see Section 3.3). In order to broaden our understanding of Uranus’s molecular distributions, it may be advantageous to focus future research efforts on the reactivity of C_2_H_2_ with other primary hydrocarbons. Improving our understanding of the chemistry of this compound, a precursor to polyynes, cyanopolyynes, and potentially PAHs, may facilitate the future detection of novel species [144].

As seen in Section 2.4 and Figure 5, the net end result of CH_4_ photolysis due to UV photons results in high Z hydrocarbons (Table 4). With Titan and Uranus having reducing atmospheres, high-altitude haze formation benefits from the supply of EUV radiation which in turn heats the haze layers and creates the observed high-altitude temperature inversions (see Figure 4). These mechanisms have been well characterized on Titan and remain poorly known on Uranus [63]. In spite of the tropopause cold-trap, condensing species such as CH_4_ manage to rise into the stratosphere under unknown mechanisms [20], possibly due to moist convection. This transport brings CH_4_ to higher altitudes (an effect even more pronounced on Neptune due to stronger mixing) whereby VUV photons begin photodissociation and photoionization [20]. The most abundant photolytic product, C_2_H_6_ is important on Titan for several reasons. First, it is one of the first photochemically produced hydrocarbons and reaches ∼10^−6^ in the stratosphere (Table 4) with detections of ice spectral signatures thought to correspond to ethane ice condensation above the tropopause over the north pole [191,192]. Second, its formation involves ion-neutral reaction coupling associated with methane loss via Reaction (5). Also, once formed, neutral ethane is likely to react with C2 cations resulting in the formation of C3 and C4 ions [70], thus accelerating molecular growth. In the EUV/FUV wavelength range, C_2_H_6_ destruction mainly leads to the formation of ethylene C_2_H_4_ and two hydrogen atoms, while at longer wavelengths (>140 nm) C_2_H_4_ is formed along with H_2_, with branching ratios of 0.30 and 0.12, respectively (see Section 3.5). This is an important step since not only is ethane an important intermediate in the photochemical production of atomic and molecular hydrogen on Titan (although the majority of hydrogen is formed thanks to CH_4_ photolysis), but also because DEA of H_2_ may thus form the H^−^ anion where low-energy (4 eV) trapped electrons reside in a resonant state within H_2_ [193]. Molecular hydrogen is also efficiently produced through the photodissociation of ethylene C_2_H_4_ with UV photons between 118 and 175 nm [194,195] through the following reaction pathway:

(10) $$C_{2}H_{4}+h\nu ⟶C_{2}H_{2}+H_{2}$$

This constitutes an important reaction since (i) ethylene (first discovered by [196]) is the smallest alkene (i.e., the two C atoms are linked by a double bond) and the only photochemical hydrocarbon to never condense at Titan’s tropopause, and (ii) photolysis of C_2_H_4_ incorporates a π→π* excitation where absorbed low-energy photons (<10 eV) lead to an electronically excited state of the molecule. In this excited state, ethylene may go through photoisomerization or proton abstraction reactions, Furthermore, ion-neutral reactions play here a key role for molecular growth by producing some of the first C3 (Reaction (11)) and then C5 (Reaction (12)) ions, what has been described by [10] as a “stepping stone from methane to higher hydrocarbons”. The reactivity of C_2_H_4_ with N(^2^D), the first electronically excited state of atomic nitrogen produced by VUV photons, has been studied on Titan and plays a key role in the loss of N(^2^D) as well as the formation of nitrogenated products [197]. Overall, photochemistry is thus a fundamental mechanism to rapidly convert small molecules into larger organics.

(11) $$C_{2}H_{4}+C_{2}{H_{5}}^{+}⟶{\mathrm{CH}}_{4}+C_{3}{H_{5}}^{+}$$

(12) $$C_{2}H_{4}+C_{3}{H_{5}}^{+}⟶H_{2}+C_{5}{H_{7}}^{+}$$

#### 3.2.3. Polycyclic Aromatic Hydrocarbons: Agents of Haze Growth?

On Titan, the composition of gas phase products with masses > 78 amu, corresponding to C_6_H_6_, is still largely unknown. Data in this molecular mass regime (heavy ions and neutrals) is austere and can be summarized in three broad categories, though with no exact molecular formulae. First, aliphatic compounds that comprise C_2_H_2_ polymers, nitrogenated polymers, and aliphatic copolymers [89]. These broad mass groups (possibly up to C14) have only been detected in their positively charged form by Cassini, at altitudes where EUV radiation peaks in the ionosphere (∼1000 km). Second, PAHs and nitrogenated-PAHs have been tentatively detected, with naphthalene (C_10_H_8_^+^) and anthracene (C_14_H_10_^+^) being candidate cations [89,156]. Ion-neutral pathways are likely dominant processes for their formation. In the gas phase, VUV photons are generally needed to ionize PAHs through radiation chemistry. Recently, Ref. [198] investigated the low-temperature visible-light photoionization of PAHs trapped in crystalline ice. The authors found that trapped PAHs in low-temperature water ice had their ionization threshold lowered by 4.4 eV with respect to their neutral gas phase counterparts. This opens new venues towards the characterization of potential PAHs in the atmospheres of Titan and Uranus. Third, negatively charged compounds (anions) have long remained a core question related to the gas composition of the upper atmosphere of Titan since their discovery by [199]. More details on them are given in Section 4.

To date, neither benzene or PAHs have not been detected on Uranus (or Neptune). However, a point should be made here to address the photochemistry of C_6_H_6_ and PAHs of relevance to Uranus, through comparative planetology with Jupiter and Saturn. A search for C_6_H_6_ was conducted on all gas giants by [200] using the Infrared Space Observatory. While abundance values were determined for Jupiter and Saturn, only upper limits were provided for Uranus and Neptune. Early photochemical modeling by [140] containing only neutral-neutral reactions and Ly-α radiation originating from the LIPM did not predict the formation of C_6_H_6_ on the two ice giants, and acknowledged the need for accurate C_6_H_6_ absorption cross-sections, branching ratios at low temperature and low pressures. Loss mechanisms part of their model included C_6_H_6_ destruction by photolysis to form either a phenyl group (C_6_H_5_) or C_6_H_4_. Another important assumption, based on Jovian photochemistry, was that C_6_H_6_ in an excited “hot” state stabilizes through collisions once transported down to Jupiter’s lower stratosphere [140], although the authors also note the possibility for benzene to be produced by ion-neutral reactions in aurorae-rich regions. Putative stabilization of benzene may thus prevent the molecule from dissociating [140]. The non-detection of benzene on Uranus (or Neptune), however, has renewed interest in incorporating ion chemistry to models, particularly when using Titan as a baseline [142,146]. In [146], coupling ion-neutral pathways brought predicted mole fractions of benzene from ∼10^−13^ (neutral reactions only) to ∼10^−9^–10^−10^ (ion-neutral reactions) between 1μbar and 1 mbar in Neptune’s stratosphere. We can expect there to be similar reactions occurring in Uranus’s atmosphere, although important caveat variables exist such as (i) temperature-dependent photodissocative branching ratios, (ii) electron dissociation recombination rates, (iii) cloud microphysics and equilibrium vapor pressure measurements at low temperature, and (iv) vertical transport [115,146,201]. Furthermore, photochemical reaction pathways, either omitted from models or whose branching ratios are unknown, involving C_2_H_2_ or its derivatives, remain to be explored [144]. As pointed out in [202], laboratory measurements of electron recombination rates of C_6_H_7_^+^ by low-energy electrons (<1 eV, Reaction (13)) with a rate constant of ($\alpha =2.4\;\times \;10^{-6}$ ${\mathrm{cm}}^{3}$ $s^{-1}$ valid for electrons temperatures between 300 and 800 K) and their products are key yet unknown parameters needed to understand the formation pathways leading to benzene. Other pathways such as the trimerization Reaction (14) may also be relevant to surface or tropospheric conditions on Titan due to GCR radiation [144]. Once C_6_H_6_ is formed, PAH formation may also be possible through the ethynyl addition mechanism (Reaction (15)) for which both Titan and Uranus may provide suitable low-temperature, hydrogen-rich environments [144,203].

(13) $$C_{6}{H_{7}}^{+}+e^{-}⟶C_{6}H_{6}+H$$

(14) $$3C_{2}H_{2}+\mathrm{GCR}⟶C_{6}H_{6}$$

(15) $$C_{6}H_{6}+C_{2}H⟶C_{6}H_{5}C_{2}H+H$$

Finally, catalytic reactions on the surface of interplanetary grains with the interaction of C_2_H_2_ with SiC has been shown to be an efficient mechanism to produce cyclic and/or prebiotic molecules (see [144] for a detailed review on the matter). Delivery of SiC grains (found in meteorites and comets) and subsequent chemistry with C_2_H_2_ may lead to C_6_H_6_ formation, while silicon dicarbide c−SiC_2_ (found in the ISM) may be produced by the UV photolysis of c−SiC_2_H_2_ (Reaction (16), [144,204]). The kinetics of these reactions, and thus their capacity to act as efficient catalyzers for larger organics formation, remains an open field of study, one of interest for the cold outer solar system. More recently, quantum chemistry calculations explored gas phase cyanobenzene formation routes catalyzed by the NCN^−^ anion [205].

(16) $$c-\mathrm{Si}C_{2}H_{2}+h\nu ⟶c-\mathrm{Si}C_{2}+H_{2}$$

### 3.3. Laboratory Experiments: Simulating Atmospheric Chemistry

From the first seminal set of studies designed to mimick the UV- and magnetospheric-field of radiation of an outer solar system body (Titan), laboratory experiments have unveiled many processes involved in the formation of laboratory analogues of planetary aerosols called *tholins* [7,8]. These studies have used multiple sources of energy to simulate either the UV photon radiation reaching the upper atmosphere of Titan, or more energetic Ly-α or GCR radiation. In the former case, electrical plasma discharges have been widely used as a realistic analogue of solar radiation-induced chemistry as impact from inelastic electron collisions will dissociate the carrier gases CH_4_ and N_2_, and their overall electron energy distribution functions resembles that of the solar spectrum [102]. The electrons deposited on N_2_-based gas mixtures thus lose their energy through inelastic collisions and are able to initiate the chemistry between N_2_, CH_4_, and any other gas present in the mixture. To reproduce the more intense radiation such as Ly-α photons, GCRs, or higher-energy photons, dedicated microwave plasma sources or synchrotron facilities (for energies < 200 nm) have been utilized. A panorama of these facilities used to simulate Titan’s atmospheric chemistry, from those studying gas phase to condensed-phase chemistry, is shown in Table 6. The reader is referred to [7] for a historical overview of experimental simulations of *tholin* formation to simulate Titan’s atmosphere.

Currently, laboratory simulations of Uranus’s atmosphere are severely limited. As discussed previously, multiple parameters needed to better understand our comprehension of photochemical haze formation under low-energy (<50 eV) particles are lacking from state-of-the-art photochemical models. Such parameters and uncertainties can be corroborated by experimental simulations using analogous Titan techniques as a basis to probe the chemistry in H_2_-based environments. Laboratory experiments such as those listed in Table 6 are in an opportune time to address these knowledge gaps, whether in determining accurate reaction kinetics, branching ratios, or in studying the photochemical evolution of organic and sulfuric Uranus-relevant ices. Following the themes outlined in [20,63], more details and future perspectives are given below and in Section 5.

- *Atmospheric chemistry science:* Multiple complementary laboratory experiments (Table 6) utilizing different sources of energy (plasmas, UV lamps, high-energy synchrotron beamlines) are substantial to probe specific chemical and photoionizing processes. In particular, questions surrounding the role of ion-molecule chemistry and haze growth on Titan have significantly benefitted from laboratory studies. Coupled with in situ or ex situ analyses such as high-resolution mass spectrometry, IR spectroscopy, electron microscopy, secondary ion mass spectrometry, X-ray photoemission spectroscopy, atmospheric-pressure photoionization, just to name a few, future measurements would provide much insights into the chemical composition of Uranian *tholins* and gas phase precursors. Furthermore, laboratory characterizations of photochemical products would help support in situ measurements by a future UOP and help quantify these precursors resulting from the photodissociation of CH_4_, NH_3_, etc.
- *Cloud science:* Laboratory measurements of the physical and optical properties of any Uranian laboratory-produced aerosols would directly provide valuable information to interpret cloud observations and the modeled scattering, nucleating, and size properties of the CCN. Their properties would then help address the role and interaction of clouds with condensable species. Moreover, studies of the photochemical evolution under low-energy (as well as of much higher-energy) photon irradiation remains critically unexplored.
- *Chemical kinetics & thermodynamics:* As outlined below, kinetic rates, branching ratios, and absorption cross-sections are fundamental properties that are needed to solve model degeneracies and inaccurate abundance retrievals (Table 7). Future theoretical calculations combined with experimental measurements are much needed.

### 3.4. Condensed Phase

Ice cloud spectral signatures have been detected on both Titan and Uranus (see Section 2). The clouds in these atmospheres are tropospheric/stratospheric and can be at similar altitudes to the photochemical hazes [8,21,252]. Over more than a decade, multiple studies have investigated the low-temperature reactivity of Titan ice cloud analogues under UV photon irradiation. These studies have also measurement the fundamental properties of these Titan-relevant ices/*tholins* with no energy input, e.g., IR absorption spectroscopic [109,252] and their optical properties [253], and vapor pressures [115,254], as well as UV photon and synchrotron light irradiation (see Table 6). Reproducing stratospheric ice photochemistry has had to adapt to the ever-evolving knowledge of Titan’s stratospheric composition and seasonal variations. Nonetheless, ice mixtures have typically been formed from any of the following molecules CH_4_, C_2_H_2_, C_4_H_2_, C_6_H_6_, HCN (Table 6). The overall objective of these studies can be summarized in two broad categories: (i) reproducing spectral features seen by Cassini’s Composite Infrared Spectrometer (CIRS) in the mid- and far-IR, and (ii) measuring the spectroscopic and chemical impacts of FUV photon irradiation on these ices. In the latter case, few laboratory apparatus have explored the ageing of UV-induced photochemistry. The Physique des Interactions Ioniques et Moléculaires (PIIM) laboratory at Aix-Marseille Université has been conducting studies of the photochemical evolution of Titan-relevant organic ices for over a decade. The foundational work for these measurements was established through the VUV-induced photosynthesis of organic molecules trapped in solid argon matrices at low temperatures [255,256]. In [256], the authors used a microwave-driven hydrogen lamp which generated FUV photons with energies 3–10 eV and generating a photon fluence of ∼10^15^ photons ${\mathrm{cm}}^{2}$ $s^{-1}$ to irradiate the Ar matrix doped with a C_2_H_2_:HC_5_N mixture. The induced photochemistry resulted in the production of HC_7_N and further confirmed the possibility to generate long condensed state carbon-nitrogen chains trapped in solid Ar. Since, experiments to simulate the photochemical processing of Titan-relevant molecules at cryogenic temperatures has included C_2_H_2_ [244], C_6_H_6_ [121,246,247,257], C_4_N_2_ [104], HCN, HC_3_N, and HC_5_N [119,120], and ethyl cyanide CH_3_CH_2_CN pure ice [243]. Note in the latter case, higher energy photons (>120 nm) were used and yielded several organics such as ethyl isocyanide CH_3_CH_2_NC, vinyl cyanide CH_2_CHCN, and hydrogen cyanide HCN. Such studies not only highlight the potential for low-energy photons to initiate solid-state chemistry in lower atmospheric regions where high-energy photons are rare, but also because they may help in retrieving desorption energies, photodissociation cross-sections, or the branching ratios of products resulting from UV photolysis [243]. In [178], FUV photons at >300 nm were found to initiate condensed-state photochemistry in C_4_N_2_ ices through singlet-triplet excitations (population of triplet states only) whereas at shorter wavelengths (266 nm) kinetics are faster due to singlet-singlet excitation processes [104,178]. Photon fluxes in these laboratory experiments reach ∼10^17^ photons ${\mathrm{cm}}^{2}$ $s^{-1}$ which corresponds approximately to <10 Earth years on Titan depending on the experimental irradiation integration time [178]. In addition, with much higher fluxes, synchrotron light is an extremely useful tool to explore a much wider range of energies; these studies are summarized in Table 6.

Several of the molecules mentioned hitherto posses their first excited singlet ($S_{0}-S_{1}$) or triplet ($S_{0}-T_{1}$) state thresholds in the FUV-VIS region which makes them particularly relevant study under cryogenic conditions, and relevant to the cold planetary atmospheres of the outer solar system [119]. As a result, solid-state photochemistry is permitted to occur at longer wavelengths than in the gas phase by reducing the excitation energy threshold of new electronic states. In these conditions, photochemistry initiated by low-energy (<20 eV) photons at altitudes where organic ice clouds form is plausible and may lead to pathways where ice CCN may harbor more advanced prebiotic chemistry [178]. These processes have been extensively studied in environments applicable to the ISM ([160], *and references therein*). Other ice mixtures containing more species of the CHNOPS family may for future laboratory work be relevant to incorporate and to investigate the photon- and electron-induced chemistry. For example, the radiolysis of ammonia (NH_3_) under low-energy (7 eV) electron impact resulted in the production of the nitrogen-rich hydrazine (N_2_H_4_) and diazene (N_2_H_2_) [159]. In their work, incident electrons with energies as low as 6 eV permitted excition processes leading to the production of NH_2_ radicals at 20 K while also producing the hydride anion H^−^. In parallel to photodissociation mechanisms, electronic excitation was shown to be a crucial process in the electro-processing of NH_3_ ice. While ammonia remains to be directly detected on Uranus (and importantly, as it condenses to form ammonium hydrosulfide NH_4_SH clouds, see Figure 3), its stratospheric and tropospheric photochemical evolution (along with the other CH_4_ and H_2_S clouds) persists as a mystery.

**Table 7:** Selected branching ratios (*br*) of photodissociation at Ly-α (121.6 nm) wavelengths (or in ranges including Ly-α as indicated if necessary) for CH_4_, H_2_, C_2_H_2_, C_2_H_4_, C_2_H_6_, CH_3_C_2_H, C_3_H_8_, C_4_H_2_, CH_3_CN, and H_2_S. AROM indicates a summed list of 14 neutral aromatics.

Molecule	Photochemical Products	*br*
${\mathrm{CH}}_{4}$	${\mathrm{CH}}_{3}$ + H	0.42
	^1^CH_2_ + $H_{2}$	0.48
	^3^CH_2_ + 2H	<0.1
	CH + $H_{2}$ + H or C + 2$H_{2}$	<0.1
$H_{2}$	$H_{2}*$ → $H_{2}$ + h$\nu^{\prime }$ (fluorescence)	0.8–0.9
	H + H (predissociation)	0.1–0.2
C_2_H_2_	C_2_H + H	0.3
	C_2_ + H_2_	0.1
	C_2_H_2_* → C_2_H_2_	0.6
	C_2_H_2_^+^ +e^−^	0.84 ^*a*^
C_2_H_4_	C_2_H_2_ + H_2_	0.58 ^*b*^
	C_2_H_2_ + 2H	0.42 ^*b*^
C_2_H_6_	C_2_H_4_ + H_2_	0.12
	C_2_H_4_ + 2H	0.30
	C_2_H_2_ → 2H_2_	0.25
	CH_4_ +1CH_2_	0.25
	2CH_3_	0.08
CH_3_C_2_H	C_3_H_3_ + H	0.56 ^*c*^
	C_3_H_2_ + H_2_	0.44 ^*c*^
C_3_H_8_	C_3_H_6_ + H_2_	0.34 ^*d*^
	C_2_H_6_ + 1CH_2_	0.09 ^*d*^
	C_2_H_5_ + CH_3_	0.35 ^*d*^
	C_2_H_4_ + CH_4_	0.22 ^*d*^
C_4_H_2_	C_4_H + H	0.20 ^*e*^
	2C_2_H	0.03 ^*e*^
	C_2_H_2_ + C_2_	0.10 ^*e*^
	C_4_H_2_*	0.67 ^*e*^
CH_3_CN	CH_3_ + CN	0.20 ^*f*^
	CH_2_CN + H	0.80 ^*f*^
H_2_S	H_2_ + S(1D)	<0.12 ^*g*^
AROM	C_6_H_6_ + photoproducts	0.1–0.3 ^*h*^

^*a*^ From 166 to 190 nm. ^*b*^ From 118 to 175 nm. ^*c*^ Extends up to 220 nm. ^*d*^ From 115 to 135 nm. ^*e*^ From 120 to 164 nm. ^*f*^ Below 235 nm. ^*g*^ For *λ* = 139.11 nm. ^*h*^ Model estimates from [142]. Note: Although propane C3H8 has not been detected on Uranus, it has been proposed in some photochemical models. References: [122,142,180,194,258,259,260,261,262,263,264].

### 3.5. Branching Ratios

UV wavelength-dependent branching ratios are one of the fundamental components required to accurately simulate photochemical pathways relevant to planetary atmospheres [194]. With the modern advances in quantum-chemistry calculations, these important tools can provide accurate quantum yields and branching ratios for the photodissociation of neutral molecules (see Table 7), positive ions (Table 8), and even of vibrationally excited states of hydrocarbons of relevance [265]. Although non-exhaustive, the tables below provide wavelength-dependent branching ratios for some simple hydrocarbon photolysis reactions that have been included in Titan, Uranus (and Neptune) photochemical models.

### 3.6. Dication Chemistry and Photo Double Ionization Processes

Fragmentation processes in the gas phase with respect to divalent states have recently, when investigating ionization chemistry mechanisms, gained interest. Indeed, double photoionization and photoelectron impact calculations of N_2_ for the first time by [268] in Titan’s upper atmosphere predicted an N_2_^++^ layer located at the ionospheric peak (∼1150 km, Reaction (17)). Fluorescence may even be observed for N_2_^++^ [268].

(17) $$A+h\nu ⟶A^{++}+2e^{-}$$

The double photoionization thresholds for our atoms and molecules of interests (i.e., C, N, abd N_2_) all occur at energies < 45 eV (34.4 eV for CH_4_; the reader is referred to the detailed review by [269], Table 1, on doubly charged ions in planetary atmospheres). Recently, vertical ionization pathways of bimolecular [CH_4_–N_2_]^2+^ or [CH_4_…CH_4_]^2+^ clusters have been calculated and provided key insights into the role of monovalent and divalent states of simple hydrocarbons may play in planetary environments [185,269,270,271]. In [270], ionized fragmentation processes led to monovalent [H_3_C–HN_2_]^+^ and divalent [H_4_C–N_2_]^2+^ intermediates with no energy barrier. This is an important characteristic since in its “ionized growth”, a bimolecular cluster may thus produce a stabilized intermediate having acquired enough excess energy, making it possible to overcome energy barriers, as described in [270]. The calculated mechanisms also revealed the involvement of a C−N covalent bond prior to further reactions eventually leading to the formation of the N_2_H^+^, CH_3_^+^, CH_3_N_2_^+^, and CH_2_N_2_^+^ cations. When studying [CH_4_…CH_4_]^2+^ clusters, [271] found that the cluster would stabilize into a C−C bond and thence produce some of the first light hydrocarbon cationic precursors. The role divalent ionization chemistry may thus play in Titan and other planetary atmospheric chemistry is still largely unexplored but deserve further scrutiny, particularly since the double ionization thresholds of simple hydrocarbons, relevant to reduced atmospheres, are relatively small when placed in the context of upper atmospheres where higher-energy (>40 eV) electrons and photons (VUV–X-ray) precipitate. These processes underscore the need to experimentally and computationally explore ionized chemistry induced by electron and photon impact as divalent A^++^ ionization pathways are likely to be relevant the upper planetary atmospheres as an overlooked phenomenon participating in organic growth [269]. Finally, although at much higher electron impact energies (300 eV), the C_2_H_6_^2+^ dication was found to yield H_3_^+^ by vertical ionization [185]. Whether these processes would also occur on Uranus is at present a mystery, although the Earth, Mars, Venus, and Titan are all thought to harbor these dications [269]. Future constraints on these ionization processes are very relevant also in the context of data analysis, since mission mass spectrometry instruments (e.g., INMS and CAPS on Cassini) have not had a high enough mass resolution to distinguish between very close nuclear mass defects [269]. As a result, negative ion mass spectra analysis and interpretation have, for example, traditionally relied on assuming singly charged ions when conducting energy-to-mass data analyses [199]. Such an assumption may be acceptable for low-mass species but loses its validity for larger molecular species and photochemical aerosols.

## 4. Negative Ion Chemistry and Haze Growth

### 4.1. Anions on Titan

For many years, anion chemistry had remained constrained primarily to Earth’s atmosphere where O (an electronegative species) atoms located in the ionosphere would undergo electron attachment reactions in the D-region, the lower part of the ionosphere <100 km [272]. Outside of Earth, negative ions have also been detected in comet Halley’s inner coma (e.g., [273]), in the Enceladus polar plume [274], and finally on Titan [199,275,276,277]. Early photochemical models did not include anion chemistry (e.g., [95,135]) and it would not be until the 2007 when the discovery of very large negative ions, first up to *m/z* (mass/charge) of 10,000 [199] and then *m/z* 13,800 by [276], that our understanding of haze growth on Titan expanded and enabled photochemical models to incorporate the first steps of an unexplored chemistry [129,278]. Several anion-neutral mechanisms have been incorporated into photochemical models [70]. While photoionization processes mainly lead to the formation of primary ions such as N_2_^+^, CH_3_^+^, or H, anions can form through dissociative attachment of electrons with suprathermal electrons [278,279,280] including:

(18) $$CH_{4}+e^{-}⟶C{H_{2}}^{-}+H_{2}\;(10.3\;\mathrm{eV})$$

(19) $$C_{2}H_{2}+e^{-}⟶C_{2}H^{-}+H\;(2.7\;\mathrm{eV})$$

Ion-pair formation,

(20) $$H_{2}+h\nu ⟶H^{-}+H+\;(17.3\;\mathrm{eV})$$

(21) $$CH_{4}+h\nu ⟶H^{-}+{{\mathrm{CH}}_{3}}^{+}\;(21.5\;\mathrm{eV})$$

Radiative electron attachment with thermal electrons,

(22) $$H+e_{Th}^{-}⟶H^{-}+h\nu$$

And proton abstraction,

(23) $$C_{2}H_{2}+H^{-}⟶C_{2}H^{-}+H_{2}\;(k=4.4\times 10^{-9})$$

DEA reactions are strongly dependent on accurate reaction rate coefficients and photoabsorption and photoionization cross-sections ([70], *and references therein*). For most molecules considered in Titan photochemical schemes (e.g., HCN, HC_3_N, and C_4_H_2_), DEA cross-sections reach maximum values at low energies (<7 eV) [127]. The DEA of methane is strongly cross-section-dependent, as seen in [127]. Depending on the DEA cross-sections used, even one or two orders of magnitude differences can induce large variations in calculated mole fractions [127]. Thus, reducing uncertainties in these cross-sections is fundamental since anion products will directly participate in the organics and haze growth. The production of the simple hydride anion (Reactions (20) and (24)), of prime interstellar interest [281], is strongly model-dependent and an important precursor since it drives many of the proton abstraction reactions leading to the larger anionic C2, C3, and C4 species [10,127,278,282]. Thence, even with relatively low electron affinities, species such as H^−^ may present abundances higher than expected, considering the non-negligible CH_4_ abundance in the atmosphere, generally slightly less abundant than the dominant CN^−^. While direct abundance measurements by Cassini of H^−^ were not feasible [277], the degree of competition between DEA and proton abstraction of neutrals by H^−^ remains uncertain, resulting in model-observation discrepancies [126]. Experimental measurements of H^−^ desorption from *tholin* aerosol analogues from 3 to 15 eV electron irradiation could hint at the hydride anion being a key species incorporated into the photochemical haze [283]. Table 9 shows the dominant anion production and loss pathways near Titan’s ionospheric peak at 1100 km.

(24) $${\mathrm{CH}}_{4}+e^{-}⟶H^{-}+{\mathrm{CH}}_{3}$$

Reaction pathways involving larger molecular compounds (>C3) become even more unresolved since these usually rely on slow and scant radiative attachment reactions (Reaction (25), see [284]). The study of these pathways has recently increased, benefiting from the detection of several anions in the ISM [281].

(25) $$C_{4}H+e^{-}⟶C_{4}H^{-}+h\nu$$

**Table 9:** Dominant production (first row of each species) and loss (second row of each species) mechanisms for negative ions on Titan at 1100 km inside the ionosphere. Reaction rate coefficients are given in ${\mathrm{cm}}^{3}$ $s^{-1}$.

Species	Reaction	Rates (cm^3^ s^−1^)	Ref.
H^−^	CH_4_ + e^−^ → H^−^ + CH_3_	($4.13\times 10^{-11}$)	[285]
	H^−^ + hν → H + e	($1.81\times 10^{-2}$)	Miller-threshold Law
CN^−^	H^−^ + HCN → CN^−^ + H_2_	$1.50\times 10^{-8}$	[286]
	CN^−^ + H → HCN + e	$6.30\times 10^{-10}$	[286]
C_2_H^−^	$H^{-}$ + $C_{2}$$H_{2}$ → $C_{2}$$H^{-}$ + $H_{2}$	$3.10\times 10^{-9}$	[282]
	$C_{2}$H$H^{-}$ + H → C_2_H_2_ + e	$1.60\times 10^{-9}$	[287]
C_3_N^−^	C_3_N + e → C_3_N^−^ + hν	$2.63\times 10^{-10}$	[288]
	C_3_N^−^ + H →HC_3_N	$5.4\times 10^{-10}$	[289]
C_5_N^−^	CN^−^ + HC_5_N → C_5_N^−^ + HCN	$5.4\times 10^{-9}$	Su-Chesnavich
	C_5_N^−^ + H → HC_5_N	$5.8\times 10^{-10}$	[289]
C_4_H^−^	C_4_H + e → C_4_H^−^ + hν	$1.1\times 10^{-8}$	[288]
	C_4_H^−^ + H → C_2_H_2_ + e	$8.3\times 10^{-9}$	[287]
C_6_H^−^	H^−^ + C_6_H_2_ → C_6_H^−^ + H_2_	$6.3\times 10^{-9}$	Langevin
	C_6_H^−^ + H → Products	$5.0\times 10^{-10}$	[287]
OH^−^	H^−^ + H_2_O → OH^−^ + H_2_	$4.8\times 10^{-9}$	[282]
	OH^−^ + hν → OH + e	$7.4\times 10^{-3}$	Miller-threshold Law
O^−^	H_2_O + e → O^−^ + H_2_	($6.56\times 10^{-12}$)	[290]
	O^−^ + hν → O + e	($1.04\times 10^{-2}$)	Miller-threshold Law

Other bimolecular negative ion reactions, such as associative detachment and cation+anion reactions also exist but the latter are generally less favorable than the aforementioned pathways for the formation of anions on Titan [10]. For a complete reaction network review, the reader is referred to [70]. Associative electron detachment have been considered in all recent photochemical models, and rely mainly on the presence of the two most abundant radical species [278] in Titan’s atmosphere: H and CH_3_ [70,278], following Reaction (26). There exists an important knowledge gap pertaining to these reactions and in particular rate constants of associative detachment reactions, and ion-neutral reactions with H and CH_3_ have been assumed to be the same [278]. A full bottom-up molecular growth promoted by anions is far from clear, especially given the difficulty in capturing exact ion densities with photochemical models [70]. To shed light on Titan’s anion composition, few laboratory studies have investigated both low-mass and intermediate-mass species [291,292], but it appears that nitrogenated anions (N/C > 1) could play a role in the growth of *tholins* [292,293]. These studies have suggested that certain molecular candidates may even contain more than three nitrogen atoms, relatively stable in N- or H-rich environments [294].

(26) $$A^{-}+B\to {\left(AB^{-}\right)}^{*}\to AB+e^{-}$$

### 4.2. Anions on Uranus

Negative ions in the atmosphere of Uranus have so far never been detected, let alone incorporated into photochemical models. In fact, their significance has been implicitly ruled out as early as 1977 [295], with the assumption of a very low abundance in the methyl radical CH_3_ and atomic hydrogen H (and even an exclusion of CH_3_ formation from the photochemical modeling of Neptune by [296]), thus possibly precluding efficient electron attachment reactions on Uranus. Radical chemistry is an important mechanism for the production of negative ions. The branching ratios and photoabsorption cross-sections of these radicals (and CH_4_) are, however, either unknown or not fully characterized [144]. As seen on Titan previously, changes in these parameters may induce orders of magnitude variations in calculated ion densities. Furthermore, experimental work by [175] have underscored the photolytic efficiency of VUV and Ly-α radiation to generate CH_3_ formation preferentially over the excited and ground-state of methylene ^1^CH_2_ and ^3^CH_2_, respectively. These branching ratios, if constrained accurately for the radiation conditions found at Uranus, may open a path for the formation of negative ions. Such pathways may further be possible depending on the variable solar input reaching Uranus. In addition, DEA mechanisms may also play a central role in Uranus’s atmosphere, as was unexpectedly discovered on Titan, and ion-pair formation triggered by photon or electron impact are also a possibility [281]. In the latter case (Reactions (20) and (21)), the energy threshold is close to the parent neutral’s ionization energy (∼10 eV) which would make neutrals also exposed to ion-pair UV photo-destruction. Nevertheless, a strong lack in accurate absorption cross-sections, radical recombination rates (see [297]), and branching ratios renders any photochemical modeling task difficult to assess whether negative ions on Uranus play an important role in the photochemical haze growth, as is it expected on Titan. Future work in this field is much needed.

### 4.3. Summary: Dissociative Electron Attachment (<20 eV)

As an important source of negative ions production, DEA processes remain fundamental for photochemical models in order to calculate anion abundances [70]. However, these depend upon accurate cross-section measurements which can sometimes vary from one experimental technique to another [298]. In addition, these cross-sections are only available for a small number of hydrocarbons and N-bearing molecules relevant to planetary atmospheres, relying at times on estimates of other similar-sized hydrocarbons [298]. Table 10 lists a condensed and non-exhaustive summary of negative ion fragments produced through low-energy DEA processes.

## 5. Summary: Opportunities for Future Studies

The planetary atmospheres of the cold regions of the outer solar system provide a unique natural laboratory presenting a wide array of chemical compositions and atmosphere-magnetosphere interactions. The composition of these atmospheres is also prone to the variables of seasonal changes and exogenic material precipitation, both affecting their thermal, chemical, and physical characteristics. The succession of the Pioneer, Voyager, and Cassini-Huygens missions opened new pathways towards the characterization of the gas giants while simultaneously spawning even more questions on their origins and evolution. Uranus and Neptune, the two least explored planets in the solar system, likely portray features that will ultimately improve our understanding of the formation of our solar system and that of exoplanets, too. The Cassini mission in particular has unearthed an invaluable amount of discovery at Titan and will continue to provide the community with an ameliorated description of the global atmosphere, given of wealth of non-analyzed data. With Uranus being placed at a top priority for a future flagship mission [1], the available breadth of observational, theoretical, and experimental techniques will help support the future investigation of the Uranian environment. As detailed in a recent community input poll, the interest in future Uranian studies encompasses a large scope of domains, from the study of the atmosphere, rings and satellites, the interior, and the magnetosphere [310]. This review attempts to describe the broad effects of photochemical processes induced by low-energy (<50 eV) photons and electrons on the growth of organic molecules in the gas and solid phases. Future synergistic work can help address many of the scientific questions and uncertainties laid out here, in order to better understand the formation mechanisms of organic molecules and chemically complex haze particles.

## Figures and Tables

**Table 1:** Spectral regions with associated energy (eV) and wavelength ranges.

	*Region 1*	*Region 2*	*Region 3*
	**Near/Far-UV** (400–121.6 nm)	**Lyman-**α (121.6 nm)	**EUV/VUV** (121.6–25 nm)
Energy range (eV)	3.1–10.2	10.2	10.2–49.6

**Table 2:** Planetary parameters and solar activity measured by Voyager 2 at Saturn, Titan, and Uranus.

	Mass ($M_{⊕}$)	Solar Constant (W m^−2^)	EUV Intensity (kR), 90–110 nm	T (1 bar Level) in Kelvin	Mean Molecular Weight
Titan	0.023	14.8	0.21	94	27.8
Saturn	95.2	14.8	0.21	134–145	2.0
Uranus	14.5	3.7	0.05	76–86	2.3

See [19,20,26] for references.

**Table 3:** Summary of all the directly detected neutral molecules in the atmospheres of Titan and Uranus [10,46,47,48,49,50,51,52,53,54].

Atoms	C		N		O		Other	
	**Titan**	**Uranus**	**Titan**	**Uranus**	**Titan**	**Uranus**	**Titan**	**Uranus**
1	CH_4_	CH_4_	HCN, HNC, CH_3_CN, HC_3_N, C_3_H_3_N, C_3_H_5_N, C_4_H_3_N	-	H_2_O, CO	CO	-	H_2_, H_2_S
2	C_2_H_2_, C_2_H_4_, C_2_H_6_	C_2_H_2_, C_2_H_6_	N_2_, C_2_N_2_	-	CO_2_	CO_2_	-	-
3	C_3_H_2_, C_3_H_4_, C_3_H_6_, C_3_H_8_	C_3_H_4_	-	-	-	-	-	-
4	C_4_H_2_	C_4_H_2_	-	-	-	-	-	-
5	-	-	-	-	-	-	-	-
6	C_6_H_6_	-	-	-	-	-	-	-

**Table 4:** Atmospheric gas phase composition in the stratospheres of Titan, Saturn, and Uranus.

Stratosphere				
Species	Titan	Saturn	Uranus	Ref.
CH_4_	1–2%	$4.7\times 10^{-3}$	16 ppm	[17,65,66]
C_2_H_2_	$2.97\times 10^{-6}$	$1\times 10^{-6}$	0.25 ppm	[67,68,69,70]
C_2_H_4_	$1.2\times 10^{-7}$	$5.9\times 10^{-7}$	<$2\times 10^{-14}$	[17,70,71]
C_2_H_6_	$7.3\times 10^{-6}$	$1\times 10^{-5}$	0.13 ppm	[17,67,68,69,70]
C_3_H_4_	$4.8\times 10^{-9}$	$1\times 10^{-9}$	0.36 ppb	[17,70,72]
C_4_H_2_	$1.12\times 10^{-9}$	$7\times 10^{-9}$	0.13 ppb	[17,70,72]
CO_2_	$1.1\times 10^{-8}$	$4.5\times 10^{-10}$	0.08 ppb	[17,70,73]
CO	$4.7\times 10^{-5}$	$2.5\times 10^{-8}$	6 ppb	[17,70,74]
H_2_O	$4.5\times 10^{-10}$	1.1 ppb	3.8 ppb	[20,70,75]
D/H (in H_2_/C_2_H_2_)	$2.1\times 10^{-4}$	$2.1\times 10^{-5}$	$4.4\times 10^{-5}$	[20,76,77]

Note: Only species shared in common and detected in all three atmospheres are shown here. For Titan, the averaged values were taken near the equator by CIRS [70]. Saturn measurements are approximate and derived from CIRS limb data near 400 km [69,72]. Abundances represented here are generally given at or near the mbar pressure level.

**Table 5:** Comparative general characteristics of photochemistry and radiation-induced chemistry in atmospheric and astrochemical processes.

Characteristics	Photochemistry	Radiation-Induced Chemistry	Examples
Energy source	Ly-α; UV continuum	EUV/X-rays; energetic particles	100–400 nm; secondary electrons; ions
Primary effect	Photodissociation; photoionization; electronic excitation	Ionization; radiolysis; dissociative electron attachment	${\mathrm{CH}}_{4}$ + hν → ${\mathrm{CH}}_{3}$ + H → H^−^ + CH_3_^+^
Key products	Radicals; small hydrocarbons	Ions (e.g., ${\mathrm{CH}}_{3}^{+}$); complex organics; electrons	H_2_^+^, CH_3_, CH_3_^+^, C_2_H_3_^+^, C_3_H_4_^+^
Timescales	ns–hours (daylight-driven)	fs–ns (instantaneous, flux-dependent)	$H_{2}$O → H• + OH• (spur reactions)
Temperature dependence	Strong (Arrhenius kinetics)	Weak (governed by particle flux)	CH_4_ + H → CH_3_ + H_2_
Electron transfer	Charge transfer	Ionization cascades; secondary electron emission	O^+^ + CH_4_
Quantum effects	Electronic transitions; spin-forbidden pathways	Ro-vibrational excitation; plasmon resonances (ices)	singlet-triplet absorption
Observables	Dayglow emissions; gas abundances	Auroral X-rays; Lyman-Werner band emissions; mass spectra of ices	mass spectra, IR-UV spectra
Altitude/region	Stratosphere; ionosphere (day side)	Thermosphere; polar auroral zones; interstellar ices	${\mathrm{NH}}_{3}$ + hν → ${\mathrm{NH}}_{2}$ + H
Desorption yields	Low–moderate (UV-photon dependent)	High (sputtering by >100 eV electrons)	CO + e^−^ → CO_(ads)_ → CO_(g)_
Multiphoton effects	Rare	Dominant (ionization cascades; track formation)	${\mathrm{CH}}_{4}$ → ${\mathrm{CH}}_{3}^{+}$ + e^−^ (15.6 eV)

References: [152,153,159,174,175,176,177,178].

**Table 6:** Panorama of past and current experimental facilities enabling the chemical study in the laboratory of simulating various typical energy sources reaching Titan’s atmosphere. The broad objectives for each experimental setup are also annotated. SE correspond to low-energy secondary electrons.

Category ^1^	Main Processes	Energy Source	References
Gas phase	Ionization, dissociation, radical chemistry	Plasma discharges	[206,207,208,209,210,211,212,213,214,215,216,217,218,219,220,221,222,223,224,225,226]
	Photolysis, radical, excitation, SE	FUV–Ly-α–EUV	[220,227,228,229,230,231,232,233,234,235,236,237,238,239,240]
Tholins/ice	Condensation, solid-state photochemistry, SE	FUV/VUV	[104,119,120,121,178,198,241,242,243,244,245,246,247]
Synchrotron	Ionization, dissociation, excitation, SE	EUV-VUV Target wavelength	[232,233,236,248,249,250,251]

^1^ Note: experimental needs for Uranus using similar techniques are listed below.

**Table 8:** Selected quantum yields of ion-neutral and neutral-neutral reactions relevant to Titan and Uranus.

Reaction	Photochemical Pathway	Quantum Yield (Φ)
N^+^ + CH_4_	CH_3_^+^ + NH	0.50
	CH_4_^+^ + N	0.05
	H_2_CN^+^ + H_2_	0.10
	HCN+ + NH + H	0.36
N_2_^+^ + CH_4_	CH_2_^+^ + N + H_2_	0.09
	CH_3_^+^ + N + H	0.91
	N_2_H^+^ + CH_3_	-
CH_3_N_2_^+^	N_2_CH_2_^+^ + H	0.01
**Atom**	**H production channels**	**H atom yield**
H	CH + CH_4_	1.00
	CH + C_2_H_6_	0.22
	CH + C_2_H_6_	0.14
	CH + C_3_H_8_	0.19
	CH + C_4_H_10_	0.14

References: [140,240,258,259,260,266,267]

**Table 10:** Low-energy dissociative electron attachment of 17 parent molecules relevant to the atmospheres of Titan and Uranus.

Parent	Fragment	Resonance Position (eV)	Cross-Section Peak (cm^2^)	Refs.
CH_4_	CH_2_^−^	10.4	$1.4\times 10^{-19}$	[127]
	H^−^	9.8	$1.6\times 10^{-18}$	[127]
H_2_	H^−^	4.0	$1.6\times 10^{-21}$	[299]
		14	$2.1\times 10^{-20}$	[299]
D_2_	D^−^	14.0	$5.5\times 10^{-21}$	[299]
C_2_H_2_	C_2_H^−^	2.8	$3.5\times 10^{-20}$	[300]
	C_2_^−^	8.3	$8.0\times 10^{-21}$	[300]
	H^−^	7.9	$3.9\times 10^{-20}$	[300]
C_2_H_4_	H^−^	10.5	$1.9\times 10^{-24}$	[301,302]
	CH^−^	9.8	*ion yield*	[302]
	C_2_H^−^	9.8	*ion yield*	[302]
	C_2_H_2_^−^	1.6	*ion yield*	[302]
	C_2_H_3_^−^	7.0	*ion yield*	[302]
C_2_H_6_	H^−^	9.2	*ion yield*	[301]
C_3_H_4_	C_3_H_3_^−^	3.4	$1.9\times 10^{-24}$	[303]
C_3_H_8_	H^−^	8.6	*ion yield*	[301]
C_4_H_2_	C_4_H^−^	2.5	$3.0\times 10^{-24}$	[304]
		5.3	$7.3\times 10^{-23}$	[304]
C_4_H_6_	H^−^	4.0	$1.4\times 10^{-24}$	[303]
C_6_H_2_	C_6_H^−^	2.8	$3.5\times 10^{-20}$, *est.*	[278,298,304]
HCN	CN^−^	1.9	$9.4\times 10^{-22}$	[305]
DCN	CN^−^	1.9	$3.4\times 10^{-22}$	[305]
NH_3_	H^−^	5.7	$2.3\times 10^{-18}$	[285]
	NH_2_^−^	5.9	$1.6\times 10^{-18}$	[285]
CH_2_N_2_	CN^−^	6.4	$3.9\times 10^{-20}$	[306]
C_2_H_4_N_2_	CN^−^	1.9	$3.9\times 10^{-20}$	[307]
H_2_S	HS^−^	1.6	$1.8\times 10^{-18}$	[308,309]
	S^−^	9.7	$4.4\times 10^{-19}$	[308,309]

Note: Certain experiments have provided the ion yields of fragments and these are annotated here. More DEA cross-section data can be found at the Innsbruck Dissociative Electron Attachment DataBase (IDEADB) node: https://ideadb.uibk.ac.at/, accessed on 9 June 2025.

## Abbreviations

UOP	Uranus Orbiter Probe
GCR	Galactic Cosmic Rays
UVS	Ultraviolet Spectrometer
NUV	Near Ultraviolet
FUV	Far Ultraviolet
VUV	Vacuum Ultraviolet
EUV	Extreme Ultraviolet
LIPM	Local Interplanetary Medium
ISRF	Interstellar Radiation Field
ISM	Interstellar Medium
JWST	James Webb Space Telescope
CRIR	Cosmic Ray Ionization Rate
INMS	Ion and Neutral Mass Spectrometer
CAPS	Cassini Plasma Spectrometer
SSI	Solar Spectrum Irradiance
CCN	Cloud Condensation Nuclei
DEA	Dissociative Electron Attachment
TNI	Temporary Negative Ions
